# Poly(l-proline)-Stabilized Polypeptide Nanostructures via Ring-Opening Polymerization-Induced Self-Assembly (ROPISA)

**DOI:** 10.1021/acsmacrolett.4c00400

**Published:** 2024-07-29

**Authors:** Ernesto Tinajero-Díaz, Nicola Judge, Bo Li, Thomas Leigh, Robert D. Murphy, Paul D. Topham, Matthew J. Derry, Andreas Heise

**Affiliations:** a Department of Chemistry, RCSI University of Medicine and Health Sciences, 123 St. Stephen’s Green, D02 YN77 Dublin, Ireland; b Aston Institute for Membrane Excellence, Aston University, B4 7ET Birmingham, U.K.; c Science Foundation Ireland (SFI) Centre for Research in Medical Devices (CURAM), D02 YN77 Dublin, Ireland; d AMBER, The SFI Advanced Materials and Bioengineering Research Centre, D02 YN77 Dublin, Ireland

## Abstract

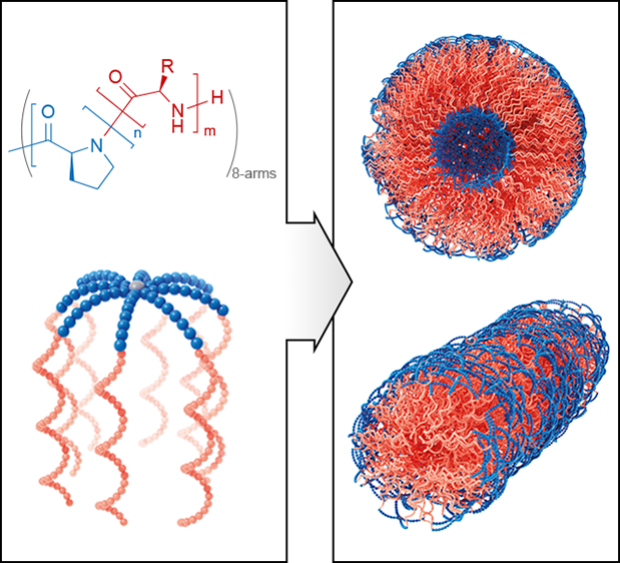

Poly(proline) II helical motifs located at the protein–water interface stabilize the three-dimensional structures of natural proteins. Reported here is the first example of synthetic biomimetic poly(proline)-stabilized polypeptide nanostructures obtained by a straightforward ring-opening polymerization-induced self-assembly (ROPISA) process through consecutive *N*-carboxyanhydride (NCA) polymerization. It was found that the use of multifunctional 8-arm initiators is critical for the formation of nanoparticles. Worm-like micelles as well as spherical morphologies were obtained as confirmed by dynamic light scattering (DLS), transmission electron microscopy (TEM), and small angle X-ray scattering (SAXS). The loading of the nanostructures with dyes is demonstrated. This fast and open-vessel procedure gives access to amino acids-based nanomaterials with potential for applications in nanomedicine.

l-Proline (LPro) stands out among the proteogenic amino acids in that it is the only amino acid with a secondary amine structure formed through a cyclic aliphatic side chain linking the α-carbon with the amino group. This unique side chain configuration imparts an unusually high level of conformational rigidity when compared to other amino acids. Longer sequences of LPro give rise to two different rigid secondary structures, which is the right-handed hydrophobic polyLPro I (PP-I) helix characterized by cis-peptide bonds and the more frequently found left-handed hydrophilic polyLPro II (PP-II) helix with trans-peptide bonds.^1^ PP-II structures are particularly abundant in globular, water dispersible proteins where they are often directly attached to helices or β strands.^2^ They are frequently found at the surface of proteins with the carbonyl and amino groups exposed to water forming regular networks of water mediated H-bonds thereby stabilizing the three-dimensional protein structures.^3,4^ Herein, we demonstrate that PP-II motifs can also stabilize synthetic peptidomimetic nanostructures obtained by open-vessel ring-opening polymerization-induced self-assembly (ROPISA).

Poly(amino acid)s are typically obtained by the ring-opening polymerization of α-amino acid *N*-carboxyanhydrides (NCA).^5,6^ Compared to other amino acids, LPro has not received much attention to date, despite the fact that its successful NCA polymerization was already reported in 1954.^7^ Several synthetic advances were since achieved, for example, the use of transition metal catalysts^8^ or methods to obtain highly pure LPro-NCA by utilizing *N*-*tert*-butyloxycarbonyl (*N*-Boc) protected LPro.^9,10^ The latter allowed the synthesis of well-defined polyLPro homopolymers and block copolymers.^11,12^ In a recent synthetic innovation Lu et al. reported water-assisted controlled polymerization of LPro-NCA in a binary acetonitrile (ACN)/water mixture affording well-defined PP-II helices.^13^ In this medium the reaction is fast, and the polyLPro remains soluble throughout, which facilitated the synthesis of high molecular weight polymers. Applying this methodology, we designed polyLPro homopolymers as ice recrystallization inhibitors mimicking ice-binding proteins commonly found in extremophile organisms^14^

We hypothesized that LPro-NCA polymerization in ACN/water could also give access to unique polyLPro nanostructures by ring-opening polymerization-induced self-assembly (ROPISA). PISA is an efficient synthesis method to prepare well-defined functional block copolymer nano-objects,^15,16^ with applications in drug delivery, cryopreservation, and stem cell storage.^17−19^ PISA dispersion polymerization involves the chain extension of a solvophilic macroinitiator (mainly in water) with a miscible monomer to yield an amphiphilic block copolymer which spontaneously self-assembles into nanostructures of different morphologies.^20−22^ While extensively demonstrated for controlled radical polymerization, combining PISA and NCA polymerization for the design of polypeptide nanostructures was only introduced recently. Due to the water sensitivity of the NCA monomers, initial procedures were carried out in organic solvents.^23−25^ Fully aqueous NCA ROPISA was achieved mitigating NCA hydrolysis by fast chain propagation.^26,27^ However, most NCA ROPISA processes reported to date rely on synthetic amino functionalized poly(ethylene glycol), PEG-NH_2_, as the solvophilic macroinitiator, which results in hybrid nanostructures. More desirable are nanostructures fully derived from amino acids for reasons of biocompatibility and degradability and as simplistic biomimetic models of natural proteins. Very recently Thornton et al. and Bonduelle et al. independently reported aqueous ROPISA using poly(sarcosine) and polyLPro as hydrophilic blocks.^28,29^ In both cases, nanostructures were obtained in a two-step process initiating NCA chain extension from preformed macroinitiators using Schlenk line and glovebox procedures. The challenge for a fully NCA ROPISA process lies in the fact that all proteogenic amino acid NCAs are hydrophobic, if not intrinsically, then through side chain protection, while a successful PISA process relies on *in situ* generation of amphiphilicity. Here we propose for the first time a straightforward procedure by which two sequential NCA polymerization steps are carried out under ROPISA conditions in a hydrophilic water-based medium in an open reactor. Employing branched polyLPro as the first solvophilic block, colloidally stable peptidomimetic nanostructures were obtained.

The synthetic methodology included three steps all carried out in an open reaction flask over a period of 5–10 min (Figure 1). In the first step, LPro-NCA was dissolved in ACN/water (1:1, v/v), which afforded a clear solution (Figure 1B-1). In the second step, a primary amine-functionalized initiator was added, resulting in the rapid formation of polyLPro and the visual release of CO_2_ (Figure 1B-2). The reaction remained clear throughout this polymerization step. Next, an NCA solution of either benzyl-l-glutamate (BLG) or carbobenzyloxy-l-lysine (ZLL) in ACN/water was added, which resulted in an opaque dispersion suggesting the formation of polypeptide particles (Figure 1B-3). Initially, hexylamine was selected as the initiator but resulted in the formation of ill-defined aggregates as well as precipitation after chain extension. This was not further optimized since better results were obtained with a multifunctional 8-arm dendritic polypropyleneimine (PPI) initiator (Figure 1). Applying the one-pot procedure, the targeted degree of polymerization (DP) of the polyLPro block was kept constant at DP = 20 per arm for all 8-arm star block copolypeptides. Disappearance of the characteristic NCA-carbonyl FTIR bands at 1789 and 1850 cm^–1^ as well as the cessation of CO_2_ evolution indicated complete conversion of the LPro-NCA after 2–3 min (Figure S3). BLG- or ZLL-NCA was then added to the clear solution targeting a DP of 10, 20, and 40 per arm, respectively (Table 1). ^1^H NMR analysis confirmed good agreement of the final block copolymer composition with the monomer feed ratio for all samples (Table 1). For example, the ^1^H NMR spectrum of PPI-(PLP_20_-*b*-PBLG_10_)_8_ exhibits characteristic signals of both polyLPro and PBLG blocks, e.g., α-COC**H**- and δ-C**H**_**2**_NH- protons at 5.25 ppm and 4.18 ppm (Figure S5). While the low solubility of the produced block copolypeptides prevented size exclusion chromatography (SEC) analysis, DOSY-NMR spectroscopy confirmed the successful chain extension (Figures S4 and S5).^14,29,30^ The DOSY-NMR spectra revealed a diffusion coefficient of 1.47 × 10^–9^ m^2^·s^–1^ for the PPI-(PP_20_)_8_. After chain extension, diffusion coefficients of all star block copolypeptides decreased in agreement with a molecular weight increase (Table 1). Notably, no residual signals of the polyLPro macroinitiator were present in the star block copolypeptide spectra, suggesting quantitative chain extension for all samples.

**Figure 1:**
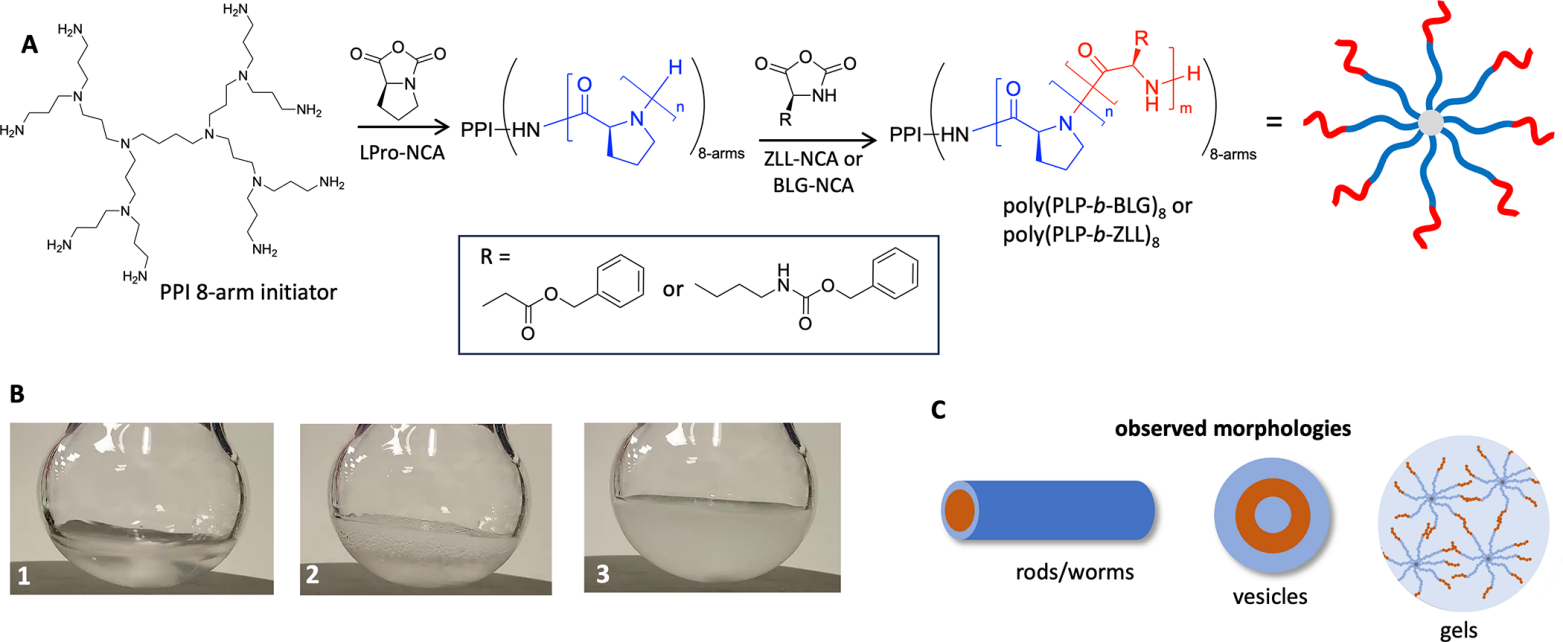
(A) One-pot synthetic route to obtain nanostructures from l-proline-based polypeptides via sequential ring-opening polymerization of α-amino acids NCA in ACN/water (1:1, v/v) at room temperature. (B) Still images showing a clear LPro-NCA solution (1), CO_2_ release upon addition of PPI initiator (2), and final opaque reaction solution after addition of BLG-NCA (3) (for full video, see Supporting Information).

**Table 1:** Results of the Synthesis of l-Proline Based Copolypeptides in ACN/Water (1:1 v/v)

copolypeptide	monomer ratios, ^1^H NMR	diff coeff, ×10^–10^ [m^2^·s^–1^]^a^	*d*_hydr_ [nm] (PDI)^b^
PPI-(PLP_20_)_8_	20/0	14.7	
PPI-(PLP_20_-*b*-PBLG_10_)_8_	21/9	2.44	60 (0.31)^c^
PPI-(PLP_20_-*b*-PBLG_20_)_8_	19/19	1.57	65 (0.29)
PPI-(PLP_20_-*b*-PBLG_40_)_8_	20/44	1.07	gel
PPI-(PLP_20_-*b*-PZLL_10_)_8_	20/10	2.51	57 (0.18)
PPI-(PLP_20_-*b*-PZLL_20_)_8_	20/22	2.45	69 (0.23)
PPI-(PLP_20_-*b*-PZLL_40_)_8_	22/48	2.13	gel

a Obtained from DOSY ^1^H NMR.

b Hydrodynamic diameter by DLS.

c Nanoparticles were nonspherical.

Following the addition of BLG- or ZLL-NCA to the polyLPro macroinitiator, samples with the longest solvophobic block, PPI-(PLP_20_-*b*-PBLG_40_)_8_ and PPI-(PLP_20_-*b*-PZLL_40_)_8_, formed gels after full NCA conversion (Figure S7). The formation of hydrogels from amphiphilic star block copolypeptides was previously observed and is a consequence of bulk physical network formation driven by hydrophobic interactions.^31^ Samples with shorter solvophobic blocks of 10 and 20 repeating units per arm transitioned from clear to opaque, and the dispersions remained stable and did not aggregate or precipitate over a period of 4 weeks (Figure S8). Dynamic light scattering (DLS, Figure S6) measurements of the colloidal dispersion revealed average particle sizes (*d*_hyd_) around 60–70 nm, which remained unchanged over the same period, corroborating the high colloidal stability of the samples. Polydispersity indices for all spherical particles were between 0.18 and 0.29, rendering the *d*_hyd_ values reliable. It was possible to remove the ACN from the dispersion by dialysis without significantly affecting the particle’s size or colloidal stability (Figure S8). Transmission electron microscopy (TEM) images (Figure 2) show worm-like structures for PPI-(PLP_20_-*b*-PBLG_10_)_8_ and spherical particles for PPI-(PLP_20_-*b*-PBLG_20_)_8_. In the case of PPI-(PLP_20_-*b*-PZLL_10_)_8_ and PPI-(PLP_20_-*b*-PZLL_20_)_8_ predominately spherical structures were seen, although a small population of worm-like structure can be identified for the former in some images.

**Figure 2:**
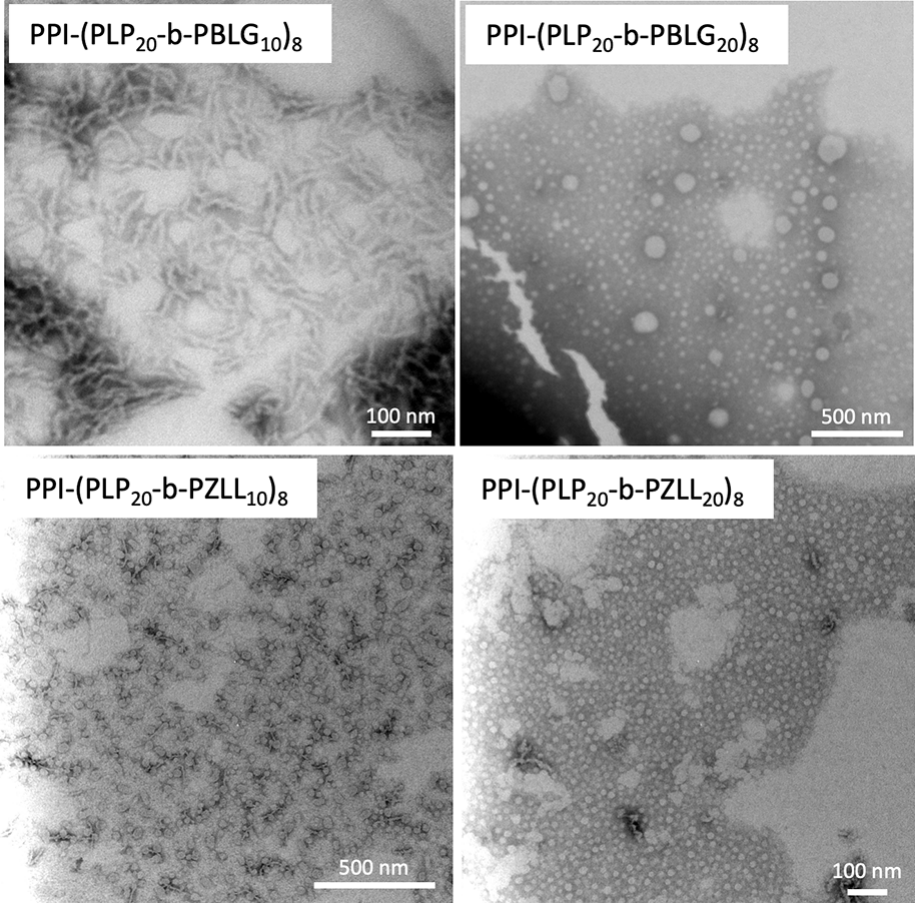
Transmission electron microscopy (TEM) images of nanoparticles from PPI-(PLP_20_-*b*-PBLG_m_)_8_ and PPI-(PLP_20_-*b*-PZLL_m_)_8_ (negative staining with1% phosphotungstic acid in water). Images were taken directly from reaction solution.

To further comprehend the TEM observations, synchrotron small-angle X-ray scattering (SAXS) experiments were conducted on 1.0% w/w dispersions in water.^32^ Inspection of the gradient of each pattern in the low *q* region of the double logarithmic plots can be used to assign the predominant copolymer morphology (Figure S9).^33,34^ A gradient of approximately −1, characteristic of worm-like micelles,^33^ was obtained for the PPI-(PP_20_-*b*-PBLG_10_)_8_ block copolypeptide dispersion, which is supported by TEM observations. Taking the local minimum in the data at *q* ∼ 0.08 Å^–1^, a worm core cross-sectional radius of 6 nm (12 nm diameter) can be approximated using *d* = 4.49/*q*,^35^ which broadly agrees with TEM analysis. As the length of PBLG block is increased, the SAXS pattern exhibits a low *q* gradient of approximately −2 that indicates the presence of flat bilayers or vesicles with thin membranes of ∼6 nm (based on a local minimum at *q* ∼ 0.08 Å^–1^).^36−38^ Unfortunately, a clear feature for the overall vesicle radius is not evident in the SAXS pattern, which may be due to a broad size distribution, as indicated by a relatively high DLS PDI value for this sample. In the case of PPI-(PLP_20_-*b*-PZLL_10_)_8_, SAXS analysis also indicated the formation of vesicle-type nanoparticles with a membrane thickness of ∼5 nm, based on the low *q* gradient of approximately −2 and the local minimum at *q* ∼ 0.09 Å^–1^, respectively. Again, a feature representative of the overall vesicle dimensions is not evident here; instead, a slight upturn in scattering intensity at low *q* suggests some aggregation of nanoparticles. Interestingly, the SAXS pattern for the PPI-(PLP_20_-*b*-PZLL_20_)_8_ dispersion exhibited a steeper low *q* gradient of approximately −4, which may suggest the formation of significantly aggregated species or poorly dispersed nano-objects. There was also an absence of a clear feature at high *q* values for this sample. While this means that SAXS cannot provide a definitive morphology assignment for PPI-(PLP_20_-*b*-PZLL_20_)_8_ nano-objects, TEM studies undoubtedly show the formation of an overall spherical morphology which we can infer are vesicular in nature because the lower molecular weight PPI-(PLP_20_-*b*-PZLL_10_)_8_ species most likely adopt this morphology. Overall, SAXS data, along with DLS measurements and TEM observations, can help to synergistically identify the most likely nanoaggregate morphologies.

To elucidate the possible polymer arrangements within the nanostructures, circular dichroism (CD) spectra were recorded for the polyLPro macroinitiator in solution and nanoparticle dispersions (Figure 3). While the interpretation of CD results from dispersed particles must be viewed with caution,^39^ some secondary structure information can be proposed. The CD spectrum of the fully soluble polyLPro macroinitiator displays a negative CD band at 206 nm and a weak positive band at 227 nm, which is characteristic of a left-handed PP-II helix.^40^ After chain extension with 10 units of BLG, the spectrum of the suspended nanoparticles shows a pattern typical of α-helices with a positive band at 193 nm and two negative bands at 198 and 208 nm ascribed to PBLG.^41^ Interestingly, the higher of the two negative bands is blue-shifted with regard to its expected position above 220 nm. This was also observed by Yang et al. for self-assembled PEG–PBLG in DMF/THF and ascribed to constricted packing of the PBLG.^42^ This constricted packing is also what we expect to observe in our system. The CD signal of α-helices often dominate CD spectra of mixed secondary structures^43^ so that it is difficult to draw conclusions about the PP-II helix in the block copolypeptides nanostructures. The shape of the α-helical trace might suggest the PP-II signal to be masked in the spectra of the block copolymers.^26^ No CD signal was recorded for particles with the longer PBLG blocks due to the low solubility of these samples. The nanoparticles obtained from the short PZLL star block copolypeptide display a CD signal resembling a β-sheet structure with a single minimum at 210 nm which is in agreement with the higher propensity of PZLL to form β-sheets at this DP.^43^

**Figure 3:**
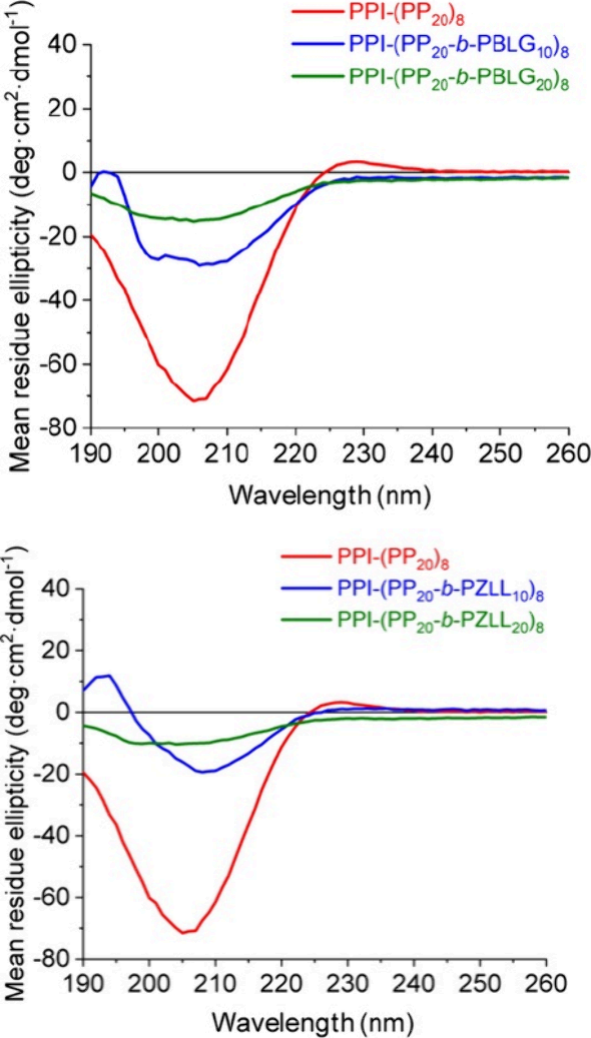
Circular dichroism (CD) spectra of PP macroinitiator and suspended star block copolypeptide nanoparticles.

The system studied here has several features that uniquely set it apart from the self-assembly of conventional amphiphilic block copolymers as well as other block copolypeptides. Both solvophilic and solvophobic blocks have a high propensity of forming rigid secondary structures that appear to be retained within the nanoparticle’s assemblies. Moreover, the solvophilic block, polyLPro, forms the inner block of the star block copolypeptide, which imposes a geometric restriction on the molecular arrangements within the nanoparticles. Taking these restrictions into account and considering the stabilizing role of PP-II at the protein–water interface of natural proteins, a self-assembly model was derived. It is reasonable to assume a similar role of the PP-II block in stabilizing the nanostructural arrangements, whereby the PP-II would form the outer, solvent exposed shell of the nanoparticles. The individual star block copolypeptides might be envisaged as a jellyfish-like conformation with the PP-II helices forming the top and the PBLG/PZLL acting as the tentacles (Figure 4A). Due to the attachment of the rigid PP-II to the central core, there must be an almost planar radial geometry of the eight PP-II arms and hence a relatively inflexible nanoparticle shell. The hydrophobic PBLG or PZLL blocks are assumed to form the core while interacting through their secondary structures, for example, helix–helix or β-sheet interactions (Figure 4B–D). Specifically the formation of anisotropic and elongated nanoparticles from amphiphilic block copolymers containing PBLG has been reported in previous studies in agreement with the structures observed here.^27−29,44,45^ It is usually ascribed to antiparallel helix–helix alignment.

**Figure 4:**
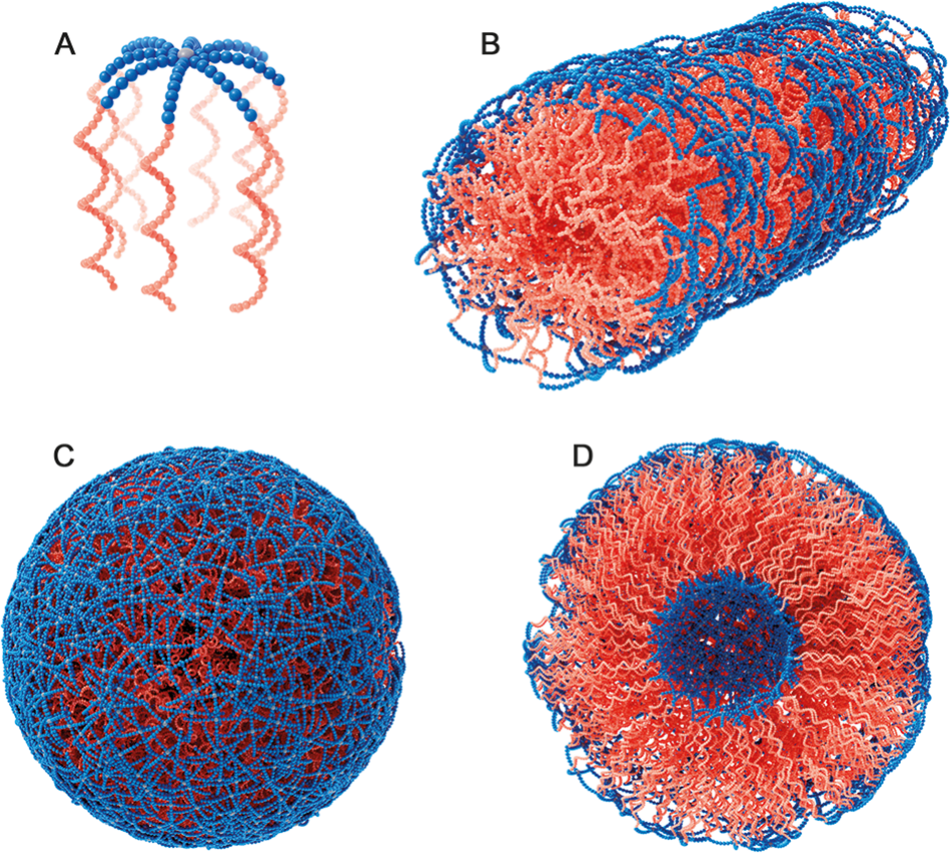
Proposed structures of nanoparticles with polyLPro (blue) and PBLG or PZLL (red) blocks: model of single 8-arm block copolypeptide structure (A), self-assembled worm-like structure (B), and spherical vesicle structures (C, D).

Preliminary experiments were carried out to investigate the ability to load the nanostructures. For PPI-(PLP_20_-*b*-PBLG_10_)_8_, Nile red (NR) and rhodamine B (RB) were selected as model dyes and added either together with the Pro NCA in the first stage of the reaction or with the BLG NCA in the second stage. After dialysis in water to remove any free dye, the dispersions appeared pink and displayed a UV absorbance in agreement with the presence of the dye (Figure S10). No significant influence on the DLS results was detected irrespective of the nature of the dye or the time point of addition, but this is only indicative due to the nonspherical morphology of the nanostructures from this sample. TEM imaging revealed retained worm-like morphologies when NR dye was used (Figure S10). It is reasonable to assume that this hydrophobic dye would localize in the hydrophobic PBLG core of the nanostructures, not influencing the overall morphology. Considering that this sample was subjected to extensive water dialysis, this speaks to the robustness of worm-like morphologies. In contrast, the more hydrophilic RB resulted in a morphology change from worm-like to mainly spherical nanostructures (Figure S11). Here the hydrophilic RB is expected to be closer to, or embedded within, the polyLPro shell, which might interrupt polyLPro interactions, causing transition to a spherical arrangement.

In conclusion, we reported a novel method for producing stable polypeptide nanoparticles using a fast open-vessel approach by sequential NCA polymerization. These nanoparticles are stabilized by PP-II secondary structures, mimicking PP-II stabilization in natural proteins. Further studies will be needed to elucidate the exact molecular interaction and the effect of composition, molecular weight, and polypeptide architecture on the nanostructure as well as their suitability as polypeptide drug delivery vehicles.

## Supporting Information Available

Experimental details, characterization of NCAs and polypeptides, DLS and stability data of nanoparticles, and SAXS plots (PDF)
Video showing clear Pro-NCA solution, CO_2_ release upon addition of PPI initiator, and final opaque reaction solution after addition of BLG-NCA (MP4)
