# Supplementary material for: Poly(l-proline)-Stabilized Polypeptide Nanostructures via Ring-Opening Polymerization-Induced Self-Assembly (ROPISA)
Source: ACS Macro Lett. 2024 Jul 29;13(8):1031–6. doi: 10.1021/acsmacrolett.4c00400 (PMC11340022; doi:10.1021/acsmacrolett.4c00400)
Supplement: Supplementary file 1 — mz4c00400_si_001.pdf [file mz4c00400_si_001.pdf]

SUPPORTING INFORMATION

Poly(L-proline) Stabilized Polypeptide Nanostructures *via* Ring-Opening Polymerization Induced Self-Assembly (ROPISA)

Ernesto Tinajero-Díaz,[a] Nicola Judge,[a] Bo Li,[a] Thomas Leigh,[a] Robert D. Murphy,[a] Paul D. Topham,[b] Matthew J. Derry,[b] and Andreas Heise*[a,c,d]

[a]Department of Chemistry, RCSI University of Medicine and Health Sciences, 123 St. Stephen's Green, D02 YN77 Dublin, Ireland. [b]Aston Institute for Membrane Excellence, Aston University, B4 7ET Birmingham, UK. [c]Science Foundation Ireland (SFI) Centre for Research in Medical Devices (CURAM), D02 YN77 Dublin, Ireland. [d]AMBER, The SFI Advanced Materials and Bioengineering Research Centre, D02 YN77 Dublin, Ireland.

Table of Contents

1 Experimental **2**

1.1 Materials 2

1.2 Methods 2

1.3 Synthesis of the α-amino acids *N*-carboxyanhydride (NCAs) 3

1.4 Synthesis of the PPI-(poly(L-proline)$_n$-*b*-poly(α-amino acids)$_m$)$_8$ PPI-(PLP$_n$-*b*-PAA$_m$)$_8$ star copolypeptide nanoparticles 4

2 Characterization **5**

3 In situ self-assembly **8**

4 Dye loading **9**

5 References **11**

1 Experimental

1.1 Materials

N-Boc-L proline (Boc-Pro) (>99.0%), γ-benzyl-L glutamate (BLG) (>99.0%), Z-L- lysine (ZLL) (98.0%) and triphosgene (99.0%) were acquired from Fluorochem Ltd. Second generation (G2) poly-propylene imine (PPI) dendrimer was obtained from SyMO-Chem BV (The Netherlands). Epichlorohydrin (ECH) (>99.0%), acetonitrile (ACN) (>99.9%), tetrahydrofuran (THF) (>99.9), ethyl acetate (>99.5%), trifluoroacetic acid (TFA) (>99.0%) were acquired from Sigma Aldrich. Chloroform-*d* ($CDCl_3$), deuterium oxide (D_2O) and trifluoroacetic acid-*d* (TFA-*d*) were purchased from Apollo Scientific. Unless otherwise noted, all reagents and chemicals were used as received without further purification.

1.2 Methods

Attenuated total reflection (ATR) FTIR measurements were performed on a Thermo Scientific Nicolet iS10 instrument. Spectra were obtained from 8 scans with a resolution of 2 cm^{-1} in the spectral region of 450–4000 cm^{-1}.

^1H and ^{13}C NMR spectra were recorded on a Bruker Avance 400 (400 MHz) spectrometer at room temperature using $CDCl_3$/TFA as solvent. All chemical shifts are reported in parts per million (ppm) and analyzed relative to the residual nuclei of $CDCl_3$ deuterated solvent.

DOSY ^1H NMR measurements were carried out on the same NMR equipment. The solvent employed was a mixture of $CDCl_3$/TFA, with the $CDCl_3$ component serving as the reference. The pulse sequence used was a longitudinal eddy current delay bipolar gradient pulse (ledbpgp2s). 104.13 gradient increments were used for each spectrum and all were acquired at a temperature of 298 K. TD = 16,384. FIDRES F2 = 0.29 FIDRES F1=250. Each spectrum was acquired with 16 scans, δ(P30) of 800 μs, Δ(D20) of 0.060 s, and a relaxation delay of 3.0 s. The maximum gradient strength was 5.35 G/mm. The DOSY spectra acquired were processed using the MestReNova 15.0.1 software and using the Bayesian transformation. Diffusion coefficients are reported in $m^2 \cdot s^{-1}$.

Dynamic light scattering (DLS) analyses were carried out using a Malvern Zetasizer Nano ZSP instrument (Malvern Instruments, Malvern UK) with a detection angle of 173° and a 3 mW He-Ne laser operating at a wavelength of 633 nm. 2.5 µL dispersed in 1 mL of deionized water of solution was used to determine size and distribution of the particles obtained.

Circular dichroism (CD) analysis of poly(proline) homopolymers and copolymers was performed on an Applied Photophysics Chirascan Plus Circular Dichroism Spectrometer at 20 °C. Copolypeptide solutions were prepared a concentration of 0.01 $mg \cdot mL^{-1}$ and analysed in

a quartz cuvette with a path length of 1 cm. Secondary structural features of recorded CD data were determined using online software BestSel.[1]

Transmission Electron Microscopy (TEM) images were recorded on a Hitachi 7650 microscope working at 120 kV. Samples were prepared by spraying a 1 mg·mL^{-1} solution of the copolypeptide onto a copper grid (200 mesh, carbon coated), dripping the water excess, and applying negative staining with an 1% phosphotungstic acid (PTA) in water.

Small-angle X-ray scattering (SAXS) patterns were recorded at a synchrotron source (Diamond Light Source, station I22, Didcot, UK;[2] Experiment ID SM33098) using monochromatic X-ray radiation (X-ray wavelength λ = 1.00 Å, with scattering vector q ranging from 0.0017 to 0.17 Å^{-1}, where $q = 4\pi \sin \theta/\lambda$ and θ is one-half of the scattering angle) and a 2D Pilatus 2M pixel detector (Dectris, Switzerland). All static SAXS measurements were performed on 1.0% w/w copolymer dispersions in 2.0 mm diameter polycarbonate capillaries. Scattering data were reduced and normalized, with glassy carbon being used for the absolute intensity calibration utilising standard routines available at the beamline.[3,4]

1.3 Synthesis of the α-amino acids *N*-carboxyanhydride (NCAs)

NCAs were synthesized following the literature procedures.[6]

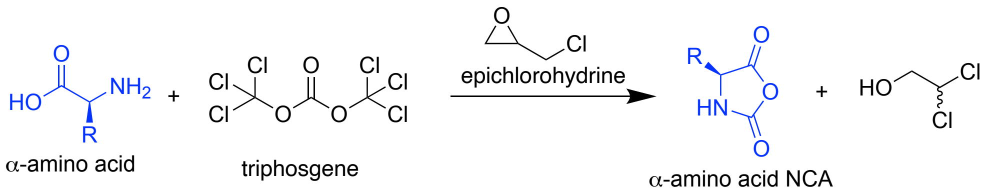

L-Proline NCA (Pro-NCA). To a round-bottom flask, *N*-Boc-L proline (10 g, 46.4 mmol, 1.0 eq), acetonitrile (150 mL), epichlorohydrin (17.1 g, 185.8 mmol, 4 eq.) were added under magnetic stirring. Afterwards, the flask was immersed in an ice bath and triphosgene (6.89 g, 23.2 mmol, 0.5 eq) was added in one portion. The reaction mixture was let to react 3 h before the filtration into a two-neck bottom flask. Then, ¾ of the ACN was removed under reduced pressure. Afterwards, and with the aid of a cannula, the mixture was precipitated in 200 mL of chilled hexane under strong stirring and kept overnight in freezer. Hexane was removed under reduced pressure and 30 mL of ethyl acetate was added to the remaining solution. Thereafter, the solution was precipitated into chilled hexane (4-fold excess) by using a cannula and let it to precipitate overnight in freezer. Subsequently, hexane was removed by using a cannula and Pro-NCA was redissolved in 30 mL of ethyl acetate, and it was precipitated in 200 mL of chilled hexane. This process was repeated twice. Finally, the purified Pro-NCA was dried overnight in a desiccator with P_2O_5 and kept sealed at -20 °C. Yield: 81%.

Pro-NCA: ^{1}H-NMR (400 MHz, $CDCl_3$, δ, ppm). 4.34 (dd, 1H), 3.73 (dt, 1H), 3.33 (ddd, 1H), 2.28 (m, 1H), 2.27 – 2.06 (m, 2H), 1.95 (m, 1H). ^{13}C-NMR (101 MHz, $CDCl_3$, δ, ppm). 168.7, 154.8, 63.0, 46.5, 27.5, 26.8.

γ-Benzyl-L glutamate NCA (BLG-NCA) and *N*$^{\varepsilon}$-carbobenzoxy L-lysine NCA (ZLL-NCA).

BLG-NCA: ^{1}H-NMR (400 MHz, $CDCl_3$, δ, ppm). 7.42-7.32 (m, 5H), 6.55 (s, 1H), 5.15 (s, 2H), 4.39 (t, 1H), 2.61 (t, 2H), 2.34-2.07 (m, 2H). ^{13}C: 173.78, 168.68, 154.38, 134.56, 67.92, 56.94, 29.60, 26.47.
ZLL-NCA: (400 MHz, $CDCl_3$/TFA-*d*, δ, ppm). 7.45-7.30 (m, 5H), 5.19 (s, 2H), 4.43 (t, 1H), 3.27 (t, 2H), 2.05-1.35 (m, 6H). ^{13}C: 169.92, 156.35, 135.88, 129.71, 69.59, 58.60, 41.63, 31.75, 29.63, 22.43.

1.4 Synthesis of the PPI-(poly(L-proline)$_n$-*b*-poly(α-amino acids)$_m$)$_8$ PPI-(PLP$_n$-*b*-PAA$_m$)$_8$ star copolypeptide nanoparticles

Synthesis of PPI-[poly(L-proline)-*b*-poly(γ-benzyl-L glutamate)]$_8$ [PPI-(PLP$_n$-*b*-PBLG$_m$)]$_8$. Pro-NCA (87.15 mg, 0.62 mmol) was dissolved in 2 mL of (ACN:water) (1:1% v/v) under magnetic stirring in a round bottom flask. Then, a solution of second generation (G2) poly-propylene imine (PPI) dendrimer as initiator, (72.2 μL, 3 mg, 0.004 mmol) in ACN:water was added into the flask ($[M]_0/[I]_0$= 20 per arm). The reaction was left to stir until the Pro-NCA had been completely consumed as monitored by ATR FTIR spectroscopy. Then, the PPI-(PLP$_{20}$)$_8$ was chain extended *via* the addition of BLG-NCA (81.1 mg, 0.308 mmol, $[M]_0/[I]_0$=10 per arm) dissolved in 4 mL of ACN:water. After full monomer conversion, the reaction mixture was dialyzed against water (MWCO = 3.5 kDa) for 24 h.

PPI-[poly(L-proline)-*b*-poly(Z-L lysine)]$_8$ [PPI-(PLP$_n$-*b*-PZLL$_m$)$_8$]. The synthesis of these copolypeptides was carried out in a similar way as for PPI-(PLP$_n$-*b*-PBLG$_m$)$_8$ copolypeptides.

2 Characterization

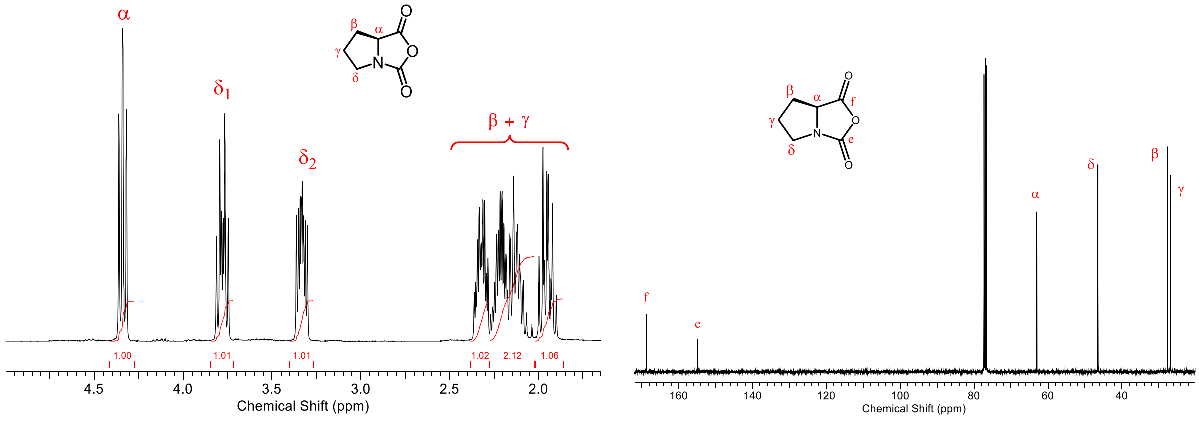

Figure S1. ^{1}H- and ^{13}C-NMR spectra of LPro-NCA (400 MHz, $CDCl_3$).

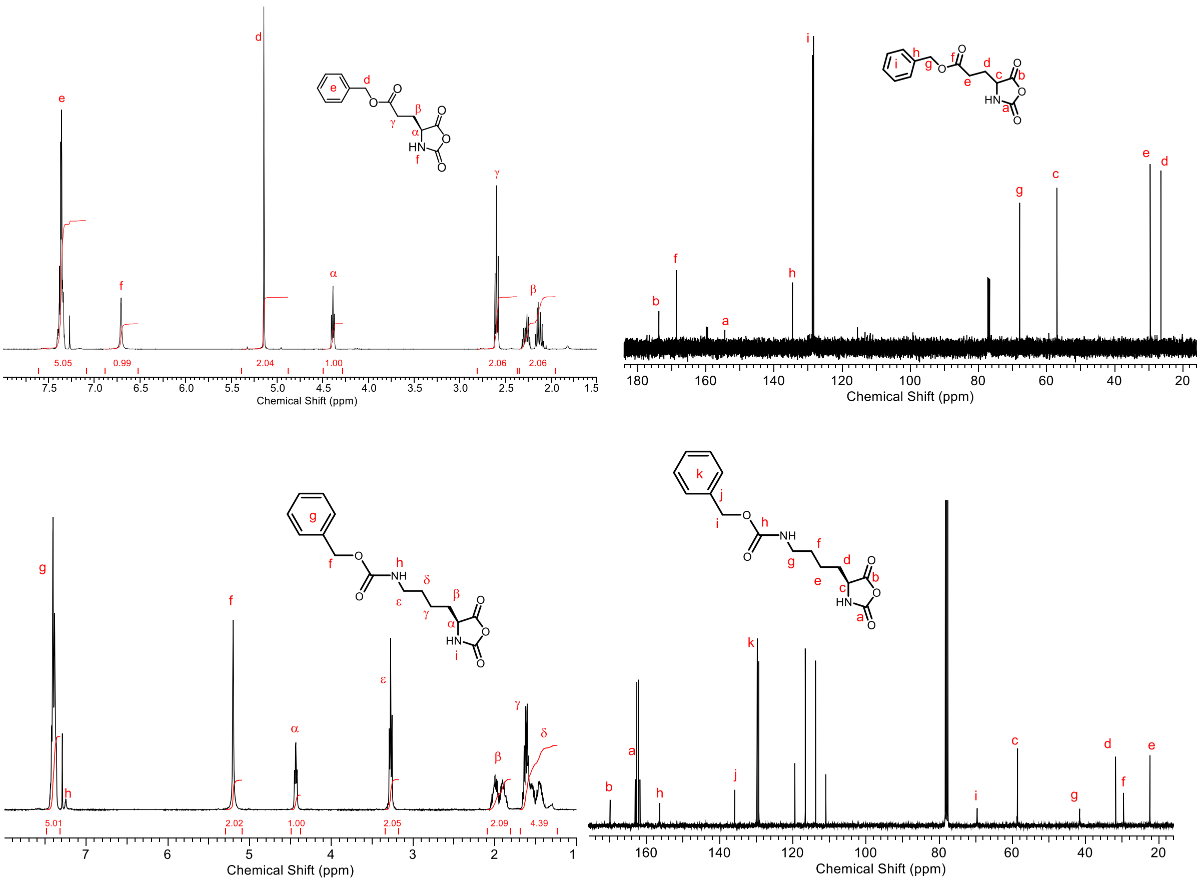

Figure S2. ^{1}H- and ^{13}C-NMR spectra of BLG-NCA (400 MHz, $CDCl_3$) and ZLL-NCA (400 MHz, $CDCl_3$/TFA-*d*).

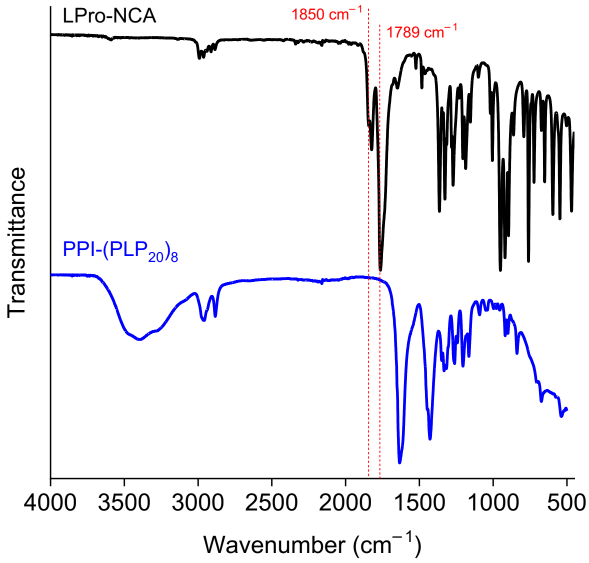

Figure S3. ATR-FTIR spectra of LPro-NCA and PPI-(PLP$_{20}$)$_8$.

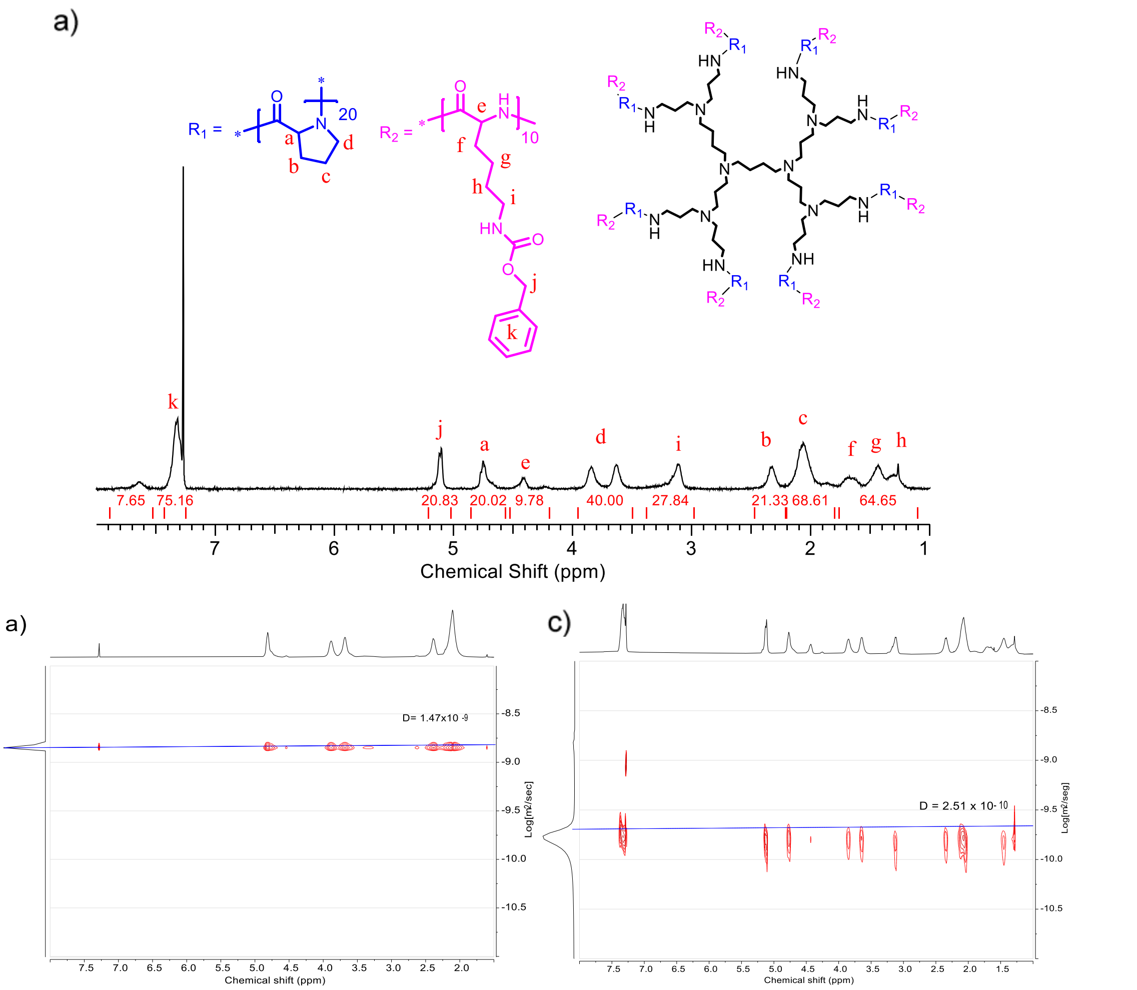

Figure S4. (a) ^{1}H-NMR (CDCl$_3$/TFA-*d*) spectrum of PPI-(PLP$_{20}$-*b*-PZLL$_{10}$)$_8$ copolypeptide, and DOSY spectra of (b) PPI-(PLP$_{20}$)$_8$ and (c) PPI-(PLP$_{20}$-*b*-PZLL$_{10}$)$_8$.

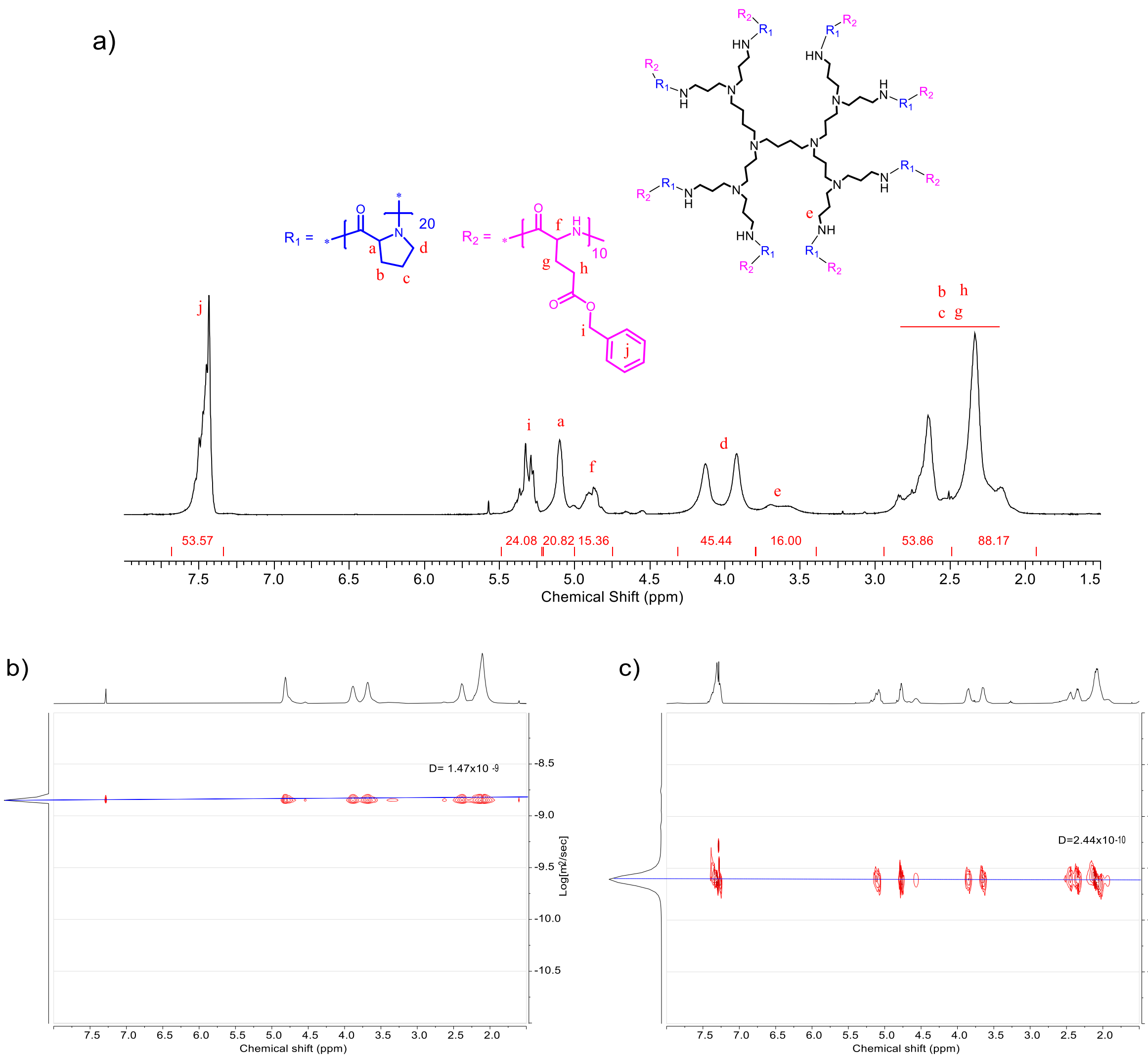

Figure S5. (a) ^{1}H-NMR ($CDCl_3$/TFA-*d*) spectrum of PPI-(PLP_{20}-*b*-$PBLG_{10}$)$_8$ copolypeptide, and DOSY spectra of (b) PPI-(PLP_{20})$_8$ and (c) PPI-(PLP_{20}-*b*-$PBLG_{10}$)$_8$.

3 *In situ* self-assembly

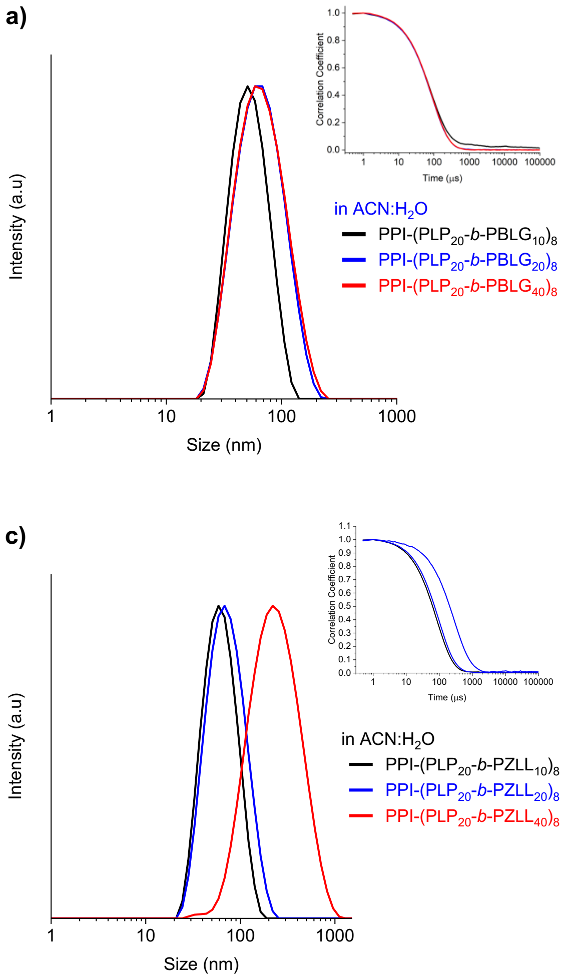

Figure S6. DLS profiles of the star-copolypeptides aggregates. Figures a), c) correspond to copolypeptides self-assembled in ANC:H_2O (1:1 v/v). Figures b), d) correspond to copolypeptides self-assembled in H_2O. Inset in each figure displays the raw-correlation-data for each copolypeptide.

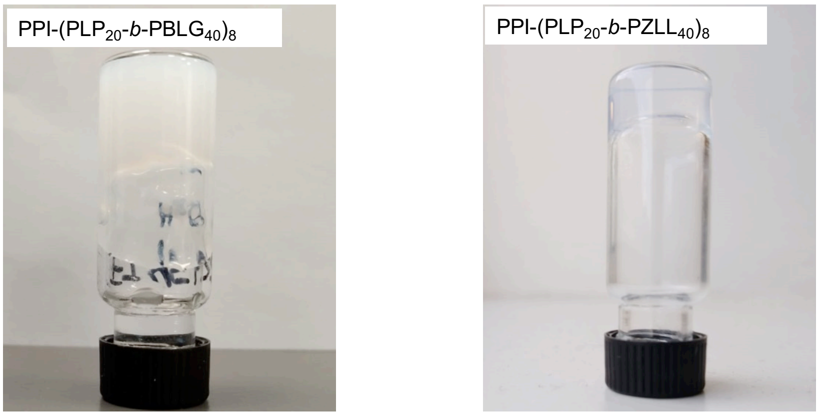

Figure S7. Inverted vials display gels formed from copolypeptide solutions.

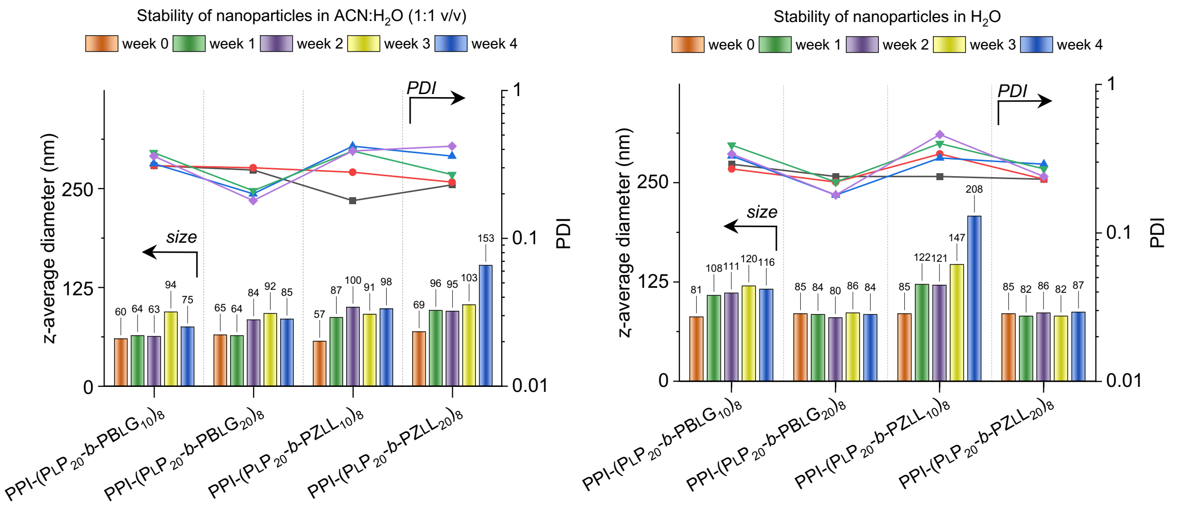

Figure S8. Stability of nanoparticles over time in ACN:H2O and H2O.

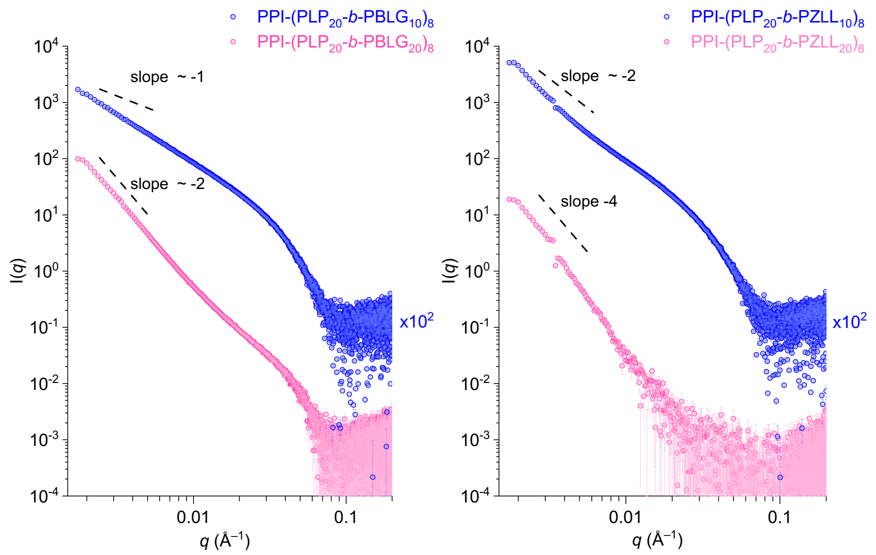

Figure S9. Small-angle X-ray scattering (SAXS) patterns for their dispersions in water. Note that blue curves are offset by two decades, *i.e.* x10^2, to ease their interpretation.

4 Dye loading

Loading of NR or RhoB in the PPI-(PLP$_{20}$-*b*-PBLG$_{10}$)$_8$ copolypeptide. For loading of either Nile Red or Rhodamine B in the first stage of the reaction, i.e. along with the Pro-NCA polymerization, dye (50 μg) and Pro-NCA (58.10 mg, 0.41 mmol) were dissolved in 1 mL of ACN:water (1:1 v/v) under magnetic stirring. Then, a solution of PPI the initiator (36.1 μL, 2 mg, 0.0026 mmol) in ACN:water was added into the flask ($[M]_0/[I]_0 = 20$). The reaction was left to stir until the Pro-NCA had been completely consumed. Then, BLG-NCA (54.18 mg, 0.206 mmol) dissolved in 2 mL of ACN:water was immediately added to the dye + PPI-(PLP$_{20}$)$_8$ mixture. After full monomer conversion, the mixture was left to stir for 4 hours. Afterwards, the reaction mixture was dialyzed (MWCO=3.5 kDa) 24 h against deionized water to

remove unloaded dye followed by DLS analysis and TEM imaging. The remainder of the sample was freeze-dried.

For loading of dye in the second stage of the reaction, i.e. along with the BLG-NCA polymerization, dye (50 μg) and BLG-NCA were mixed in ACN:water to have a dye + NCA solution. This solution was immediately added to the PPI-(PLP_{20})$_8$ macroinitiator solution. After full monomer consumption, the reaction mixture was treated as described before to have a dye + PPI-(PLP_{20}-b-$PBLG_{10}$)$_8$ conjugate.

Dye loaded polymers were assessed by UV-vis spectroscopy at a wavelength of 586 nm for Nile Red or 545 nm for Rhodamine B, respectively.

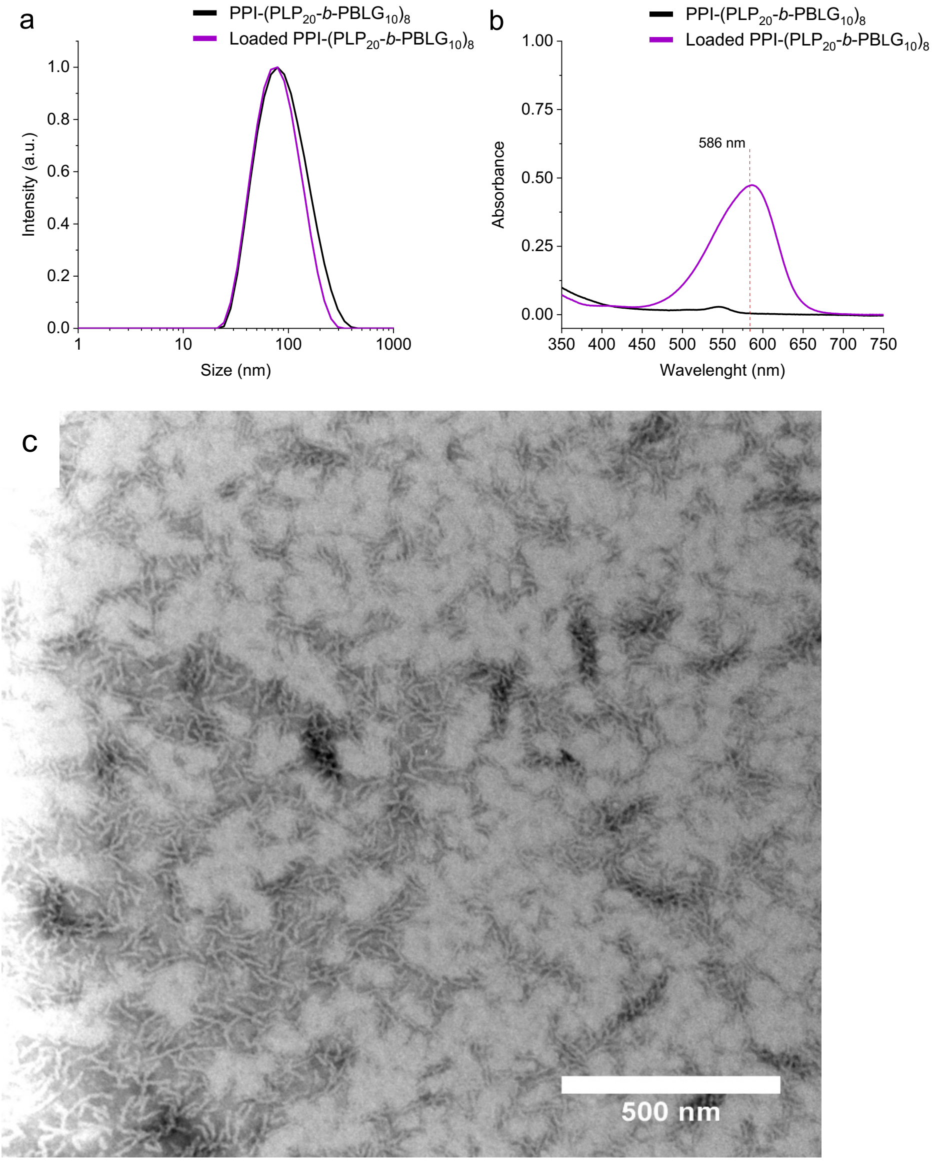

Figure S10. Nile Red loaded nanoparticles from PPI–(PLP_{20}-*b*-$PBLG_{10}$)$_8$. (a) DLS profiles, (b) UV-vis spectra and (c) TEM image of the loaded structures.

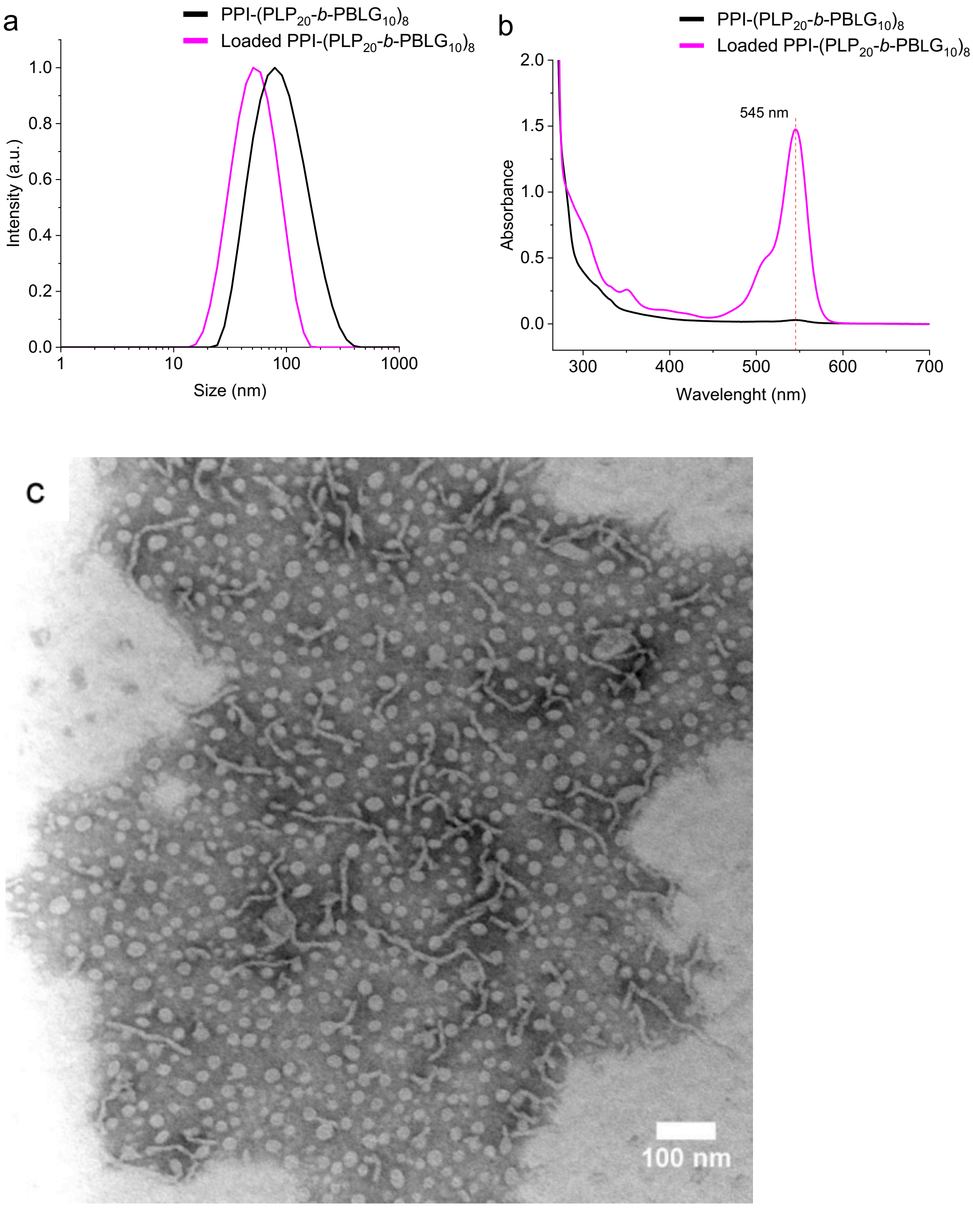

Figure S11. Rhodamine B loaded nanoparticles from PPI–(PLP$_{20}$-*b*-PBLG$_{10}$)$_8$. (a) DLS profiles, (b) UV-vis spectra and (c) TEM image of the loaded structures.

5 References

[1] A. Micsonai, F. Wien, L. Kernya, Y.-H. Lee, Y. Goto, M. Réfrégiers, J. Kardos, *Proc. Natl. Acad. Sci. U. S. A.* **2015**, *112*, E3095-103.

[2] A. J. Smith, S. G. Alcock, L. S. Davidson, J. H. Emmins, J. C. Hiller Bardsley, P. Holloway, M. Malfois, A. R. Marshall, C. L. Pizzey, S. E. Rogers, O. Shebanova, T. Snow, J. P. Sutter, E. P. Williams, N. J. Terrill, *J. Synchrotron Radiat.* **2021**, *28*, 939–947.

[3] B. R. Pauw, A. J. Smith, T. Snow, N. J. Terrill, A. F. Thünemann, *J. Appl. Crystallogr.* **2017**, *50*, 1800–1811.

[4] J. Filik, A. W. Ashton, P. C. Y. Chang, P. A. Chater, S. J. Day, M. Drakopoulos, M. W. Gerring, M. L. Hart, O. V. Magdysyuk, S. Michalik, A. Smith, C. C. Tang, N. J. Terrill, M. T. Wharmby, H. Wilhelm, *J. Appl. Crystallogr.* **2017**, *50*, 959–966.

[5] K. Manalastas-Cantos, P. V. Konarev, N. R. Hajizadeh, A. G. Kikhney, M. V. Petoukhov, D. S. Molodenskiy, A. Panjkovich, H. D. T. Mertens, A. Gruzinov, C. Borges, C. M. Jeffries, D. I. Svergun, D. Franke, *J. Appl. Crystallogr.* **2021**, *54*, 343–355.

[6] Z.-Y. Tian, Z. Zhang, S. Wang, H. Lu, *Nat. Commun.* **2021**, *12*, 5810.
